# The association between stress and mood across the adult lifespan on default mode network

**DOI:** 10.1007/s00429-016-1203-3

**Published:** 2016-03-12

**Authors:** José Miguel Soares, Paulo Marques, Ricardo Magalhães, Nadine Correia Santos, Nuno Sousa

**Affiliations:** 1 Life and Health Sciences Research Institute (ICVS), School of Health Sciences, University of Minho, Campus Gualtar, 4710-057 Braga, Portugal; 2 ICVS/3B’s, PT Government Associate Laboratory, Braga, Guimarães, Portugal; 3 Clinical Academic Center - Braga, Braga, Portugal

**Keywords:** Aging, Stress, Mood, Default mode network, Connectivity

## Abstract

Aging of brain structure and function is a complex process characterized by high inter- and intra-individual variability. Such variability may arise from the interaction of multiple factors, including exposure to stressful experience and mood variation, across the lifespan. Using a multimodal neuroimaging and neurocognitive approach, we investigated the association of stress, mood and their interaction, in the structure and function of the default mode network (DMN), both during rest and task-induced deactivation, throughout the adult lifespan. Data confirmed a decreased functional connectivity (FC) and task-induced deactivation of the DMN during the aging process and in subjects with lower mood; on the contrary, an increased FC was observed in subjects with higher perceived stress. Surprisingly, the association of aging with DMN was altered by stress and mood in specific regions. An increased difficulty to deactivate the DMN was noted in older participants with lower mood, contrasting with an increased deactivation in individuals presenting high stress, independently of their mood levels, with aging. Interestingly, this constant interaction across aging was globally most significant in the combination of high stress levels with a more depressed mood state, both during resting state and task-induced deactivations. The present results contribute to characterize the spectrum of FC and deactivation patterns of the DMN, highlighting the crucial association of stress and mood levels, during the adult aging process. These combinatorial approaches may help to understand the heterogeneity of the aging process in brain structure and function and several states that may lead to neuropsychiatric disorders.

## Introduction

Magnetic resonance imaging (MRI) studies, including volumetric, diffusion and functional analysis, have provided new insights into the structural and functional modulation of brain regions and networks across the lifespan. Among them, resting state functional MRI is being widely used to assess brain regional interactions that comprise the resting state networks (RSNs). Probably, the most explored RSN is the default mode network (DMN) (Raichle et al. 2001; Andrews-Hanna et al. 2010; Buckner et al. 2008). The DMN is thought to serve important cognitive functions such as supporting internal mental activity based on personal introspection, autobiographical memories, and thoughts of the future, playing also a role in connecting internal and external attention in monitoring the world around us (Greicius et al. 2003a; Mason et al. 2007; Giugni et al. 2010; Andrews-Hanna 2011). Although the DMN typically refers to functionally connected brain regions during the resting state, there are also relevant insights about its deactivation during attention-demanding tasks (Fox et al. 2005; Arbabshirani et al. 2012; Landin-Romero et al. 2015; Hansen et al. 2014; Anticevic et al. 2012). Based on this “deactivation” during such demanding tasks, the DMN is also referred to as the “task-negative network”. Interestingly, abnormal patterns of DMN FC and deactivation have been associated with several mental conditions, disease states and neuropsychiatric disorders (Whitfield-Gabrieli and Ford 2012; De Vogelaere et al. 2012; Zhu et al. 2012; Sampaio et al. 2013; Landin-Romero et al. 2015).

Normal brain aging is a continuous process characterized by a pattern of selective loss and preservation of structures and functions (Meunier et al. 2014; Dennis and Cabeza 2008). The central trend regarding age-induced alterations in resting-state FC is a widespread reduction throughout the brain during the lifespan that has been more commonly reported after 60 years of age for the DMN (Damoiseaux et al. 2008; Mowinckel et al. 2012; Huang et al. 2015; Marstaller et al. 2015). Additionally, task-induced deactivation studies have also reported impaired DMN deactivation in older adults (Grady et al. 2006; Harrison et al. 2011; Hafkemeijer et al. 2012).

There are some consistent, although quite limited, reports on changes in brain regions implicated in the maladaptive stress response and decreased mood (as already described by our group; Soares et al. 2014); importantly, a larger extent occurs in similar brain regions. In fact, prolonged stress exposure impairs spatial working memory, perceptual attention, behavioral flexibility and decision making both in rodents and humans in association with structural changes in several brain regions that are common to the DMN (Yuen et al. 2012; Dias-Ferreira et al. 2009; Soares et al. 2012). Importantly, most of these maladaptive structural and functional responses to increased chronic stress were reported in young and middle-aged subjects and were in part shown to be reversible (Bian et al. 2012; Soares et al. 2012; Sousa et al. 2000). Moreover, we have recently shown that stressed subjects present increased activation of the DMN, associated with impairments in the deactivation pattern and paralleled by a network volumetric atrophy (Soares et al. 2013a) that is recovered after a stress-free period in young individuals (Soares et al. 2013b). Emerging findings from major depression studies have pointed to altered connectivity and abnormal deactivation patterns in the DMN. Despite the discrepancy, the major trend suggests an increased FC in depressed subjects in DMN anterior regions, particularly in young subjects (Zhu et al. 2012; Kerestes et al. 2013); however, several studies have also reported decreased connectivity in DMN and other RSNs in the elderly (Wu et al. 2011; Veer et al. 2010; Andreescu et al. 2011), as well as a failure in DMN deactivation during task performance (Sheline et al. 2009) and even no differences between controls and depressed subjects (Sexton et al. 2012).

One of the hallmarks of healthy aging is its variability, which arises from the experience of multiple factors across the lifespan, among which exposure to stressful experience and variations in mood are major factors. Understanding the association between structural and functional brain alterations, in combination with life experiences, across the lifespan, may help to understand part of this variability, namely in cognitive performance. Herein, we address this interaction using a neuroimaging approach to assess the individual and interactive association between aging, stress, mood and the DMN, both during rest and task-induced deactivation. Based on the described individual association of aging, stress and mood on brain DMN, we hypothesize that the interaction between stress and mood (at different levels) throughout adult life is correlated with the typical DMN patterns and may help to clarify the heterogeneity of the aging process. Specifically, we expect a significant interaction of higher stress (based on our previous studies) and mood levels on the normal aging DMN connectivity decrease pattern, especially due to their potential opposite effects.

## Materials and methods

### Ethics statement

The study was conducted in accordance with the principles expressed in the Declaration of Helsinki and was approved by the Ethics Committee of Hospital de Braga (Portugal). The study goals and tests were explained to all participants and all gave informed written consent.

### Participants and psychological tests

A sample of 120 participants, selected from a representative sample of the Portuguese population in terms of age, gender and education (Santos et al. 2013), was assessed. A team of certified psychologists performed a neuropsychological test battery, which comprised: the Mini-Mental State Examination (MMSE) (Folstein et al. 1975) to determine general cognitive status; the Perceived Stress Scale (PSS) (Cohen et al. 1983), a questionnaire used to assess perceived stress in the last month, with higher scores indicating higher levels of perceived stress; the Geriatric Depression Scale (GDS, long version; higher scores indicate a more depressed mood) (Yesavage et al. 1982), to assess mood in the past recent months; and, a laterality test. Exclusion criteria included inability to understand the informed consent, participant choice to withdraw from the study, incapacity and/or inability to attend the MRI session, dementia and/or diagnosed neuropsychiatric and/or neurodegenerative disorder (medical records). From the original sample, nine subjects refused to undergo MRI screening at the time of the evaluation, four subjects had brain lesions/pathology and three subjects were excluded due to head motion. In total, 104 subjects were included in the analysis.

### Data acquisition

Participants were scanned on a clinically approved Siemens Magnetom Avanto 1.5 T (Siemens Medical Solutions, Erlangen, Germany) MRI scanner on Hospital de Braga using a Siemens 12-channel receive-only head coil. The imaging sessions included one structural T1-weighted and two functional T2*-weighted acquisitions (one resting state and one task related), conducted on the same day. For structural analysis, a T1 high-resolution anatomical sequence, 3D MPRAGE (magnetization prepared rapid gradient echo) was performed with the following scan parameters: repetition time (TR) = 2.730 s, echo time (TE) = 3.48 ms, 176 sagittal slices with no gap, flip angle (FA) = 7°, in-plane resolution = 1.0 × 1.0 mm^2^ and slice thickness = 1.0 mm. During the resting-state fMRI acquisition, using gradient echo-weighted echo-planar images (EPIs), the participants were instructed to keep their eyes closed and to think about nothing in particular. The imaging parameters were: 180 volumes, TR = 2 s, TE = 30 ms, FA = 90°, in-plane resolution = 3.5 × 3.5 mm^2^, 30 interleaved slices, slice thickness = 4 mm, imaging matrix 64 × 64 and FoV = 224 mm. The fMRI task was acquired using the same parameters as the resting state protocol, except the number of volumes, 456 in this case. The functional paradigm was presented using the fully integrated and synchronized fMRI system IFIS-SA.

### Image pre-processing

Before any data processing and analysis, all acquisitions were visually inspected by a certified neuroradiologist and confirmed that they were not affected by critical head motion and that participants had no brain lesions.

To achieve signal stabilization and allow participants to adjust to the scanner noise, the first five fMRI volumes (10 s) were discarded. For both resting state and task analysis, data preprocessing was performed using SPM8 (Statistical Parametrical Mapping, version 8, http://www.fil.ion.ucl.ac.uk) analysis software. Images were firstly corrected for slice timing using the first slice as reference and SPM8’s Fourier phase shift interpolation. To correct for head motion, images were realigned to the mean image with a six-parameter rigid-body spatial transformation and estimation was performed at 0.9 quality, 4 mm separation, 5 mm full-width at half-maximum (FWHM) smoothing kernel and using second-degree B-Spline interpolation. For resting state analysis, seven subjects were excluded once their head motion was higher than 2 mm in translation or 1° in rotation. Head movements were also included as nuisance covariates. Images were then spatially normalized with a non-linear transformation to the Montreal Neurological Institute (MNI) standard coordinate system using SPM8 EPI template and trilinear interpolation. Data were then re-sampled to 3 × 3 × 3 mm^3^ using sinc interpolation, smoothed to decrease spatial noise with an 8 mm, FWHM, Gaussian kernel. Resting state images were then temporally band-pass filtered (0.01–0.08 Hz, to reduce the effect of very low- and high-frequency physiological noise) and the linear trend was removed, and fMRI task images were high-pass temporal filtered (filter width of 128 s).

### Structural analysis

The DMN structural analysis based on segmentation of brain cortical and subcortical structures from T1 high-resolution anatomical data was performed using the Freesurfer toolkit version 5.1 (http://surfer.nmr.mgh.harvard.edu). Intracranial volume (ICV) was used to correct the regional volumes and the processing pipeline was the same as previously described (Soares et al. 2012). DMN was defined by the summed volume of the angular gyrus of the inferior parietal lobe, the posterior cingulate, the precuneus and the frontopolar region (Raichle et al. 2001; Buckner et al. 2008).

### Independent component analysis and identification of DMN

Independent component analysis was conducted using the Group ICA 2.0d of fMRI Toolbox (GIFT, http://www.icatb.sourceforge.net) (Correa et al. 2005; Calhoun et al. 2001). Briefly, spatial ICA analysis is a fully data-driven approach that consists in extracting the non-overlapping spatial maps with temporally coherent time courses that maximize independence. The GIFT workflow can be summarized in three main stages: dimensionality reduction, estimation of the group independent components and back-reconstruction of each subject’s corresponding independent components. The reduction of dimensionality of the functional data and computational load was performed with principal component analysis (PCA). Then, 20 independent components were estimated, based on a good trade-off (clustering/splitting) between preserving the information in the data while reducing its size (Zuo et al. 2010; Beckmann et al. 2005), using the iterative Infomax algorithm. The ICASSO tool was used to assess the ICA reliability, and 20 computational runs were performed on the dataset, during which the components were being recomputed and compared across runs (Himberg et al. 2004). The previous steps resulted in the estimation of a mixing matrix with partitions, unique to each subject. The individual independent components were then back-reconstructed from the group-level components. This back-reconstruction step is accomplished by projecting each subject’s data onto the inverse of the partition of the calculated matrix corresponding to that subject. The obtained independent components were expressed in *t* statistic maps, which were finally converted to a *Z* statistic. *Z* statistic describes the voxels that contributed more intensely to a specific independent component, providing a degree of FC within the network (Bartels and Zeki 2005; Beckmann et al. 2005). The final components were visually inspected, sorted and spatially correlated with resting state functional networks from Shirer et al. (2012). Each subject’s map corresponding to the best-fit component of the DMN was used to perform second-level statistical analyses. With this analysis, it is possible to identify which regions (belonging to the DMN) present an increased/decreased FC relative to the global DMN signal.

### RSN deactivation during fMRI task analysis

The *n*-back task, a standard working memory measure in cognitive neuroscience and in fMRI (Owen et al. 2005), was used to investigate the task-induced deactivations. In this study, a modified version with a block design was used, containing four different conditions: rest, 0-back, 1-back and 2-back. Four blocks of each condition were presented, randomly distributed along the experiment. Each block was composed of an instruction card (6000 ms) that indicated the condition of the block, followed by 16 trial cards and a pause card (10,000 ms) at the end. In each trial card a letter was presented for 500 ms (letters included were b/c/d/p/t/w) followed by a fixation cross presented for 2000 ms. Responses were allowed during the presentation of the letter and trial cards. In rest blocks, trial cards consisted in consecutive presentations of the letter ‘X’ and participants were instructed to remain still. In the 0-back blocks (control condition), the instruction card presented a target letter and participants were instructed to press a button whenever the trial card presented the same letter as the target letter. In 1-back (low working memory load condition) blocks, participants were instructed to press the button whenever the trial card letter matched the letter presented one trial earlier. In 2-back blocks (high working memory load condition), participants were instructed to press the button whenever the trial card letter matched the letter presented two trials earlier. Each block of the 0-back, 1-back and 2-back conditions had four target trials. At the first-level, one general linear model (GLM) was set modeling the four conditions individually (i.e., one regressor for each condition) with blocks of 40 s duration and onset on the first trial card of the corresponding block. Additionally, seven regressors of no interest were included: one modeling the instruction and pause cards and six corresponding to the six motion parameters estimated during preprocessing. One contrast of interest was defined as rest > (0-back + 1-back + 2-back)/3, thus revealing task-induced deactivations. The resulting functional patterns were masked with the DMN mask (Shirer et al. 2012). For this analysis, only 35 participants were included and the criteria used for inclusion was more than 50 % of correct responses.

### Statistical analyses

For both structural [using the IBM SPSS Statistics software, v.22 (IBM, New York)] and functional analysis (using the SPM8 software), multiple regressions were performed modeling the effects of age, gender, PSS and GDS as well as the age × PSS, age × GDS and age × PSS × GDS interactions, all included in the same model. In the structural analysis, ICV was also included to control for different head sizes, while in the functional analyses gray matter volume was included as a regressor, as older individuals present consistently higher gray matter atrophy, with potential impact on decreased brain activity (Damoiseaux et al. 2008; Mowinckel et al. 2012). For each positive or negative correlation, the results were controlled for the other covariates. The key assumptions for multivariate linear regression analysis were met and the covariates were mean-centered to avoid multicollinearity issues (Aiken and West 1991; Frazier et al. 2004).

DMN volumetric analyses were considered significant at *p* < 0.05. Functional analyses were performed using the second-level random effect analyses in SPM8. Initially, analyses were performed only to confirm the FC of the DMN, using one-sample *t* tests. Thereafter, multiple regression analysis was performed and results were considered significant at *p* < 0.05 corrected for multiple comparisons using the Monte Carlo correction. The correction was determined over 1000 Monte Carlo simulations using AlphaSim program distributed with REST software tool (http://resting-fmri.sourceforge.net/) with the following input parameters: individual voxel probability threshold = 0.025, cluster connection radius = 3 mm, Gaussian filter width (FWHM) = 8 mm, number of Monte Carlo simulations = 1000 and mask set to the DMN template mask. Anatomical labeling was defined by a combination of visual inspection and anatomical automatic labeling atlas (AAL) (Tzourio-Mazoyer et al. 2002).

The significant two-way interactions were further investigated fitting three additional models in which the PSS scores or GDS scores were centered: (1) one standard deviation (SD) below their mean, (2) at the mean and (3) one SD deviation above the mean while maintaining the remaining regressors mean centered. This enabled the investigation of how different PSS or GDS scores modulated the aging effect on brain functional patterns/volumetry. Similarly, for the three-way interactions, the age effect on RSNs functional patterns and volumetry was assessed by simultaneously centering the PSS and GDS scores: (1) both one SD deviations below their means; (2) the first variable one SD below and the second one SD above the respective means; (3) the first one SD above and the second one SD below its mean; and (4) both one SD above the respective means.

## Results

Descriptive statistics of the participants are presented in Table 1. Age was not correlated with PSS (*r* = −0.0249, *p* = 0.8084) or GDS scores (*r* = −0.0574, *p* = 0.5767).

**Table 1:** Descriptive statistics of the study participants

Characteristics	Mean (SD)	Range
Males/females	52/52	–
Age (years)	65.20 (8.07)	51–82
Education (years)	5.43 (3.84)	0–12
MMSE score	26.66 (3.30)	20–30
PSS	21.49 (8.18)	7–48
GDS	10.91 (6.70)	0–27

### Individual association of age, stress and mood with the DMN

**Fig. 1:**
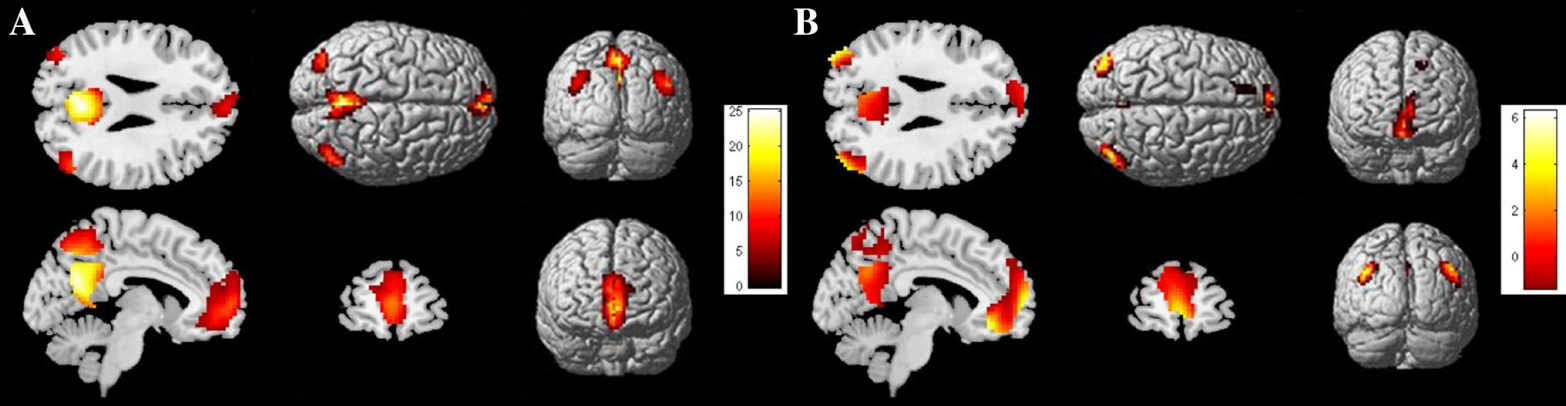
Global patterns of default mode network functional connectivity at rest (**a**) and during task-induced deactivation (**b**)

**Fig. 2:**
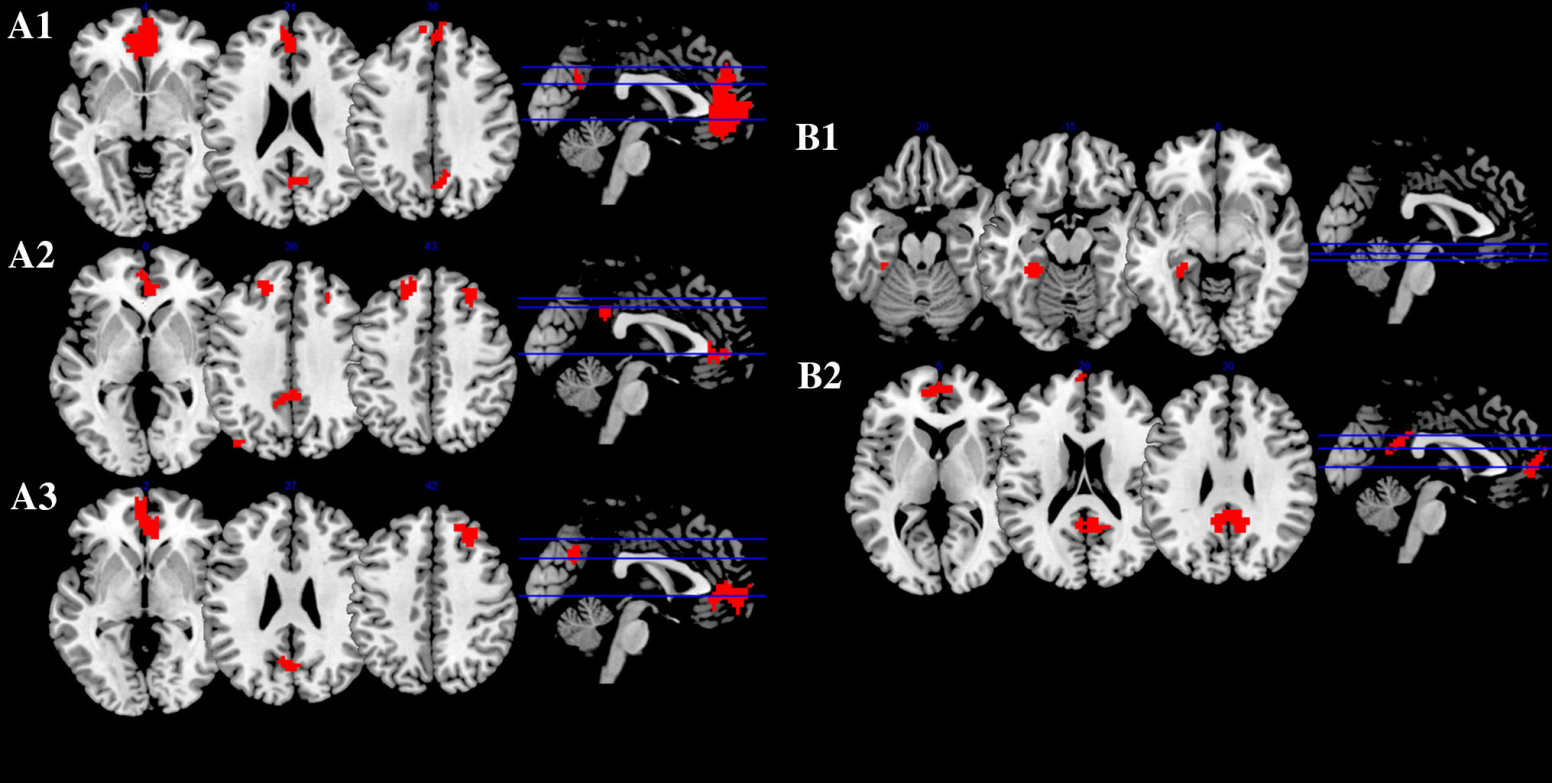
Individual association of age (**a1**, negative correlation), stress (**a2**, positive correlation) and mood (**a3**, negative correlation) with DMN during resting state, and during DMN task-induced deactivations with age (**b1**, negative correlation) and mood (**b2**, negative correlation)

The patterns of FC of the typical DMN regions during resting state (Fig. 1a) and the DMN task-induced deactivation pattern (Fig. 1b) were initially confirmed. At rest, increased age was significantly correlated with less FC in the DMN, specifically in the right anterior cingulate cortex, frontal medial orbitofrontal and precuneus relative to the normal global DMN signal (Table 2; Fig. 2a1). During task-induced deactivations, a negative correlation between age and functional deactivation was found in the left fusiform of the DMN (Table 3; Fig. 2b1). A negative correlation tendency between the DMN volume and age was observed (*T* = −4.1226, *p* = 0.16).

**Table 2:** Effect of age, stress and mood on resting-state default mode network connectivity (multiple regressions, corrected for multiple comparisons, *p* < 0.05)

Effect	Correlation	Regions	Peak MNI coordinates	Cluster size (voxels)	Maximum *Z* score
Age	Negative	Anterior cingulate cortex (right)	3, 42, 3	601	3.98
		Frontal medial orbitofrontal cortex (right)	3, 54, 0		3.41
		Precuneus (right)	12, −63, 24	145	3.61
PSS	Positive	Frontal superior gyrus (left)	−21, 39, 42	63	3.34
		Frontal middle gyrus (right)	30, 36, 45	52	3.25
		Middle cingulate gyrus (left)	−12, −45, 33	87	2.94
		Posterior cingulate gyrus (right)	6, −39, 30		2.87
		Precuneus (right)	12, −45, 6	63	2.68
		Occipital middle gyrus (left)	−36, −87, 27	68	2.57
		Frontal medial orbitofrontal cortex (left)	−3, −51, −3	52	2.55
GDS	Negative	Frontal middle gyrus (right)	27, 39, 42	69	3.68
		Cuneus (left)	−6, −66, 24	59	3.29
		Calcarine (left)	−9, −63, 15		2.61
		Frontal medial orbitofrontal cortex (left)	−3, 60, −9	154	2.66
		Anterior cingulate cortex (left)	−3, 54, 0		2.64
		Frontal medial orbitofrontal cortex (right)	9, 39, −6		2.60
		Parietal inferior lobule (right)	57, −60, 42		2.59
		Frontal middle gyrus (right)	39, 15, 60	60	3.09
		Frontal middle gyrus (left)	−36, 15, 39	55	2.99

**Table 3:** Effect of age, stress and mood on default mode network task-induced deactivations (multiple regressions, corrected for multiple comparisons, *p* < 0.05)

Effect	Correlation	Regions	Peak MNI coordinates	Cluster size (voxels)	Maximum *Z* score
Age	Negative	Fusiform (left)	−30, −40, −14	60	2.84
GDS	Negative	Precuneus (right)	18, −49, 16	177	2.78
		Posterior cingulate cortex (left)	−9, −46, 25		2.74
		Anterior cingulate cortex (left)	0, 62, 13	64	2.55
		Frontal superior medial gyrus (right)	3, 53, 1		2.52
		Frontal superior medial gyrus (left)	−3, 62, 16		2.49

Regarding stress, as measured by PSS scores, a positive correlation with FC during rest was found in the left frontal superior gyrus and medial orbitofrontal cortex, middle cingulate gyrus, occipital middle regions and in the right frontal middle gyrus, posterior cingulate cortex and precuneus of the DMN (Table 2; Fig. 2a2). Interestingly, there was also a trend for a negative correlation between the volume of the DMN and PSS scores (*T* = −1.5556, *p* = 0.12).

As for mood, assessed by GDS scores, a negative correlation with FC was found during resting state, in the DMN, particularly in the right frontal middle gyrus, left cuneus, calcarine, anterior cingulate cortex and in the bilateral frontal medial orbitofrontal cortex (Table 2; Fig. 2a3). During task performance, the DMN (in the right precuneus, left posterior and anterior cingulate cortices and bilateral frontal superior medial gyrus) deactivated less in subjects with higher GDS scores (Table 3; Fig. 2b2). No correlation between GDS scores and DMN volumetry was found (*T* = 0.6703, *p* = 0.50).

### Association of the interactions age × stress and age × mood with the DMN

**Fig. 3:**
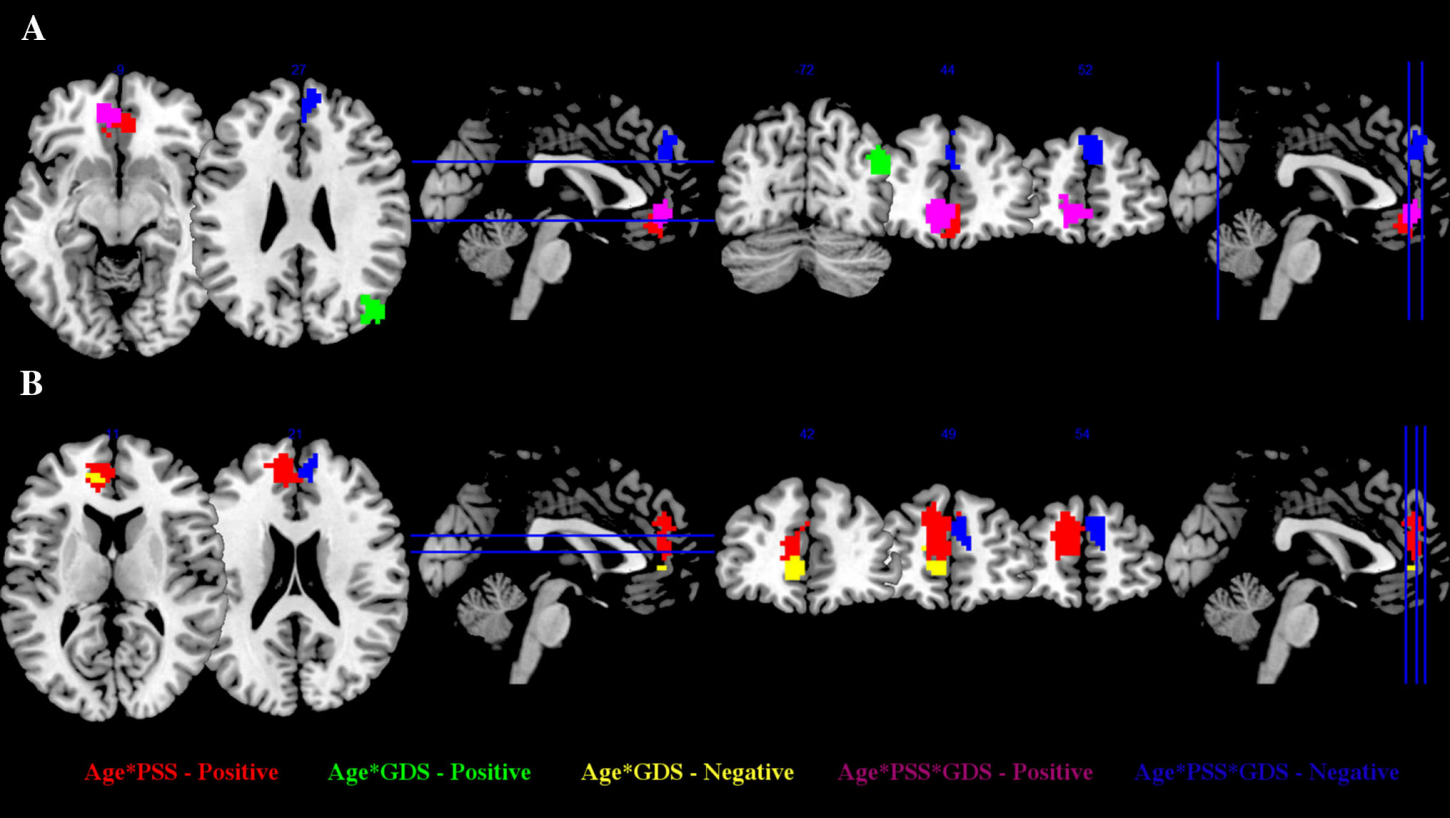
DMN regions presenting significant interactions of age × PSS positive (in *red*), age × GDS positive (in *green*) and negative (in *yellow*) and age × PSS × GDS positive (in *violet*) and negative (in *blue*), during resting state (**a**) and task-induced deactivations (**b**)

Next, we assessed the interaction of age with each of the factors under scrutiny in this work. During rest, specifically in the right frontal middle gyrus, for low and medium PSS scores, the FC decreased with age; however, in subjects with high PSS scores an opposite pattern of increased connectivity was found (Table 4; Fig. 3a). No correlation was observed concerning DMN volume and the interaction between age and PSS scores (*T* = 0.0715, *p* = 0.94).

**Table 4:** Effect of the interaction between stress, mood and aging on the default mode network functional connectivity (multiple regressions, corrected for multiple comparisons, *p* < 0.05)

Interaction	Correlation	Regions	Peak MNI coordinates	Cluster size (voxels)	M − SD/M/M + SD	Maximum *Z* score
Age × PSS	Positive	Frontal middle gyrus (right)	3, 39, −15	78	−0.0683/−0.0120/0.0290	3.46
Age × GDS	Positive	Temporal middle gyrus (right)	42, −66, 21	76	−0.0414/−0.0078/0.0258	2.92

Interaction	Correlation	Regions	Peak MNI coordinates	Cluster size (voxels)	LPSS_LGDS/LPSS_HGDS/HPSS_LGDS/HPSS_HGDS	Maximum *Z* score
Age × PSS × GDS	Positive	Frontal medial orbitofrontal cortex (left)	−6, 45, −9	135	0.0102/−0.0513/−0.0734/0.0275	3.57
		Anterior cingulate cortex (right)	9, 33, 3			2.73
	Negative	Frontal superior medial gyrus (right)	6, 48, 33	96	−0.0627/0.0191/0.0217/−0.0544	3.88

*M* mean, *SD* standard deviation, *LPSS* low Perceived Stress Scale scores, *HPSS* high Perceived Stress Scale scores, *LGDS* low Geriatric Depression Scale scores, *HGDS* high Geriatric Depression Scale scores

As for the interaction of age with GDS scores, in the right temporal middle gyrus, the FC decreased with age at low and medium scores in the GDS, but the opposite was found for subjects with higher GDS scores (Table 4; Fig. 3a). No correlation between this interaction and DMN volumetry was found (*T* = 0.3244, *p* = 0.75).

In task performance, significant correlations with the DMN deactivation were found. Specifically, in the bilateral frontal superior gyrus, for low PSS scores the deactivation decreased with aging, while for higher scorers an opposite pattern (increased deactivation) was observed. Regarding the DMN deactivation (particularly, in the left anterior cingulate cortex) for the interaction between age and GDS scores, an increased deactivation was observed with age for low and medium GDS scores; however, at high GDS scores the DMN deactivation decreases with age (Table 5; Fig. 3b).

**Table 5:** Effect of the interaction between stress, mood and aging on DMN task-induced deactivation (multiple regressions, corrected for multiple comparisons, *p* < 0.05)

Interaction	Correlation	Regions	Peak MNI coordinates	Cluster size (voxels)	M − SD/M/M + SD	Maximum *Z* score
Age × PSS	Positive	Frontal superior gyrus (right)	6, 47, 34	296	−0.0104/0.0066/0.0237	2.72
		Frontal superior gyrus (left)	−12, 50, 25			2.62
Age × GDS	Negative	Anterior cingulate cortex (left)	−9, 44, 1	98	0.0177/0.0007/−0.0164	2.89

Interaction	Correlation	Regions	Peak MNI coordinates	Cluster size (voxels)	LPSS_LGDS/LPSS_HGDS/HPSS_LGDS/HPSS_HGDS	Maximum *Z* score
Age × PSS × GDS	Negative	Frontal superior medial gyrus (right)	12, 53, 22	62	−0.0262/0.0193/0.0425/0.0048	2.70

*M* mean, *SD* standard deviation, *LPSS* low Perceived Stress Scale scores, *HPSS* high Perceived Stress Scale scores, *LGDS* low Geriatric Depression Scale scores, *HGDS* high Geriatric Depression Scale scores

### Combined interactions between age × PSS × GDS with the DMN

**Fig. 4:**
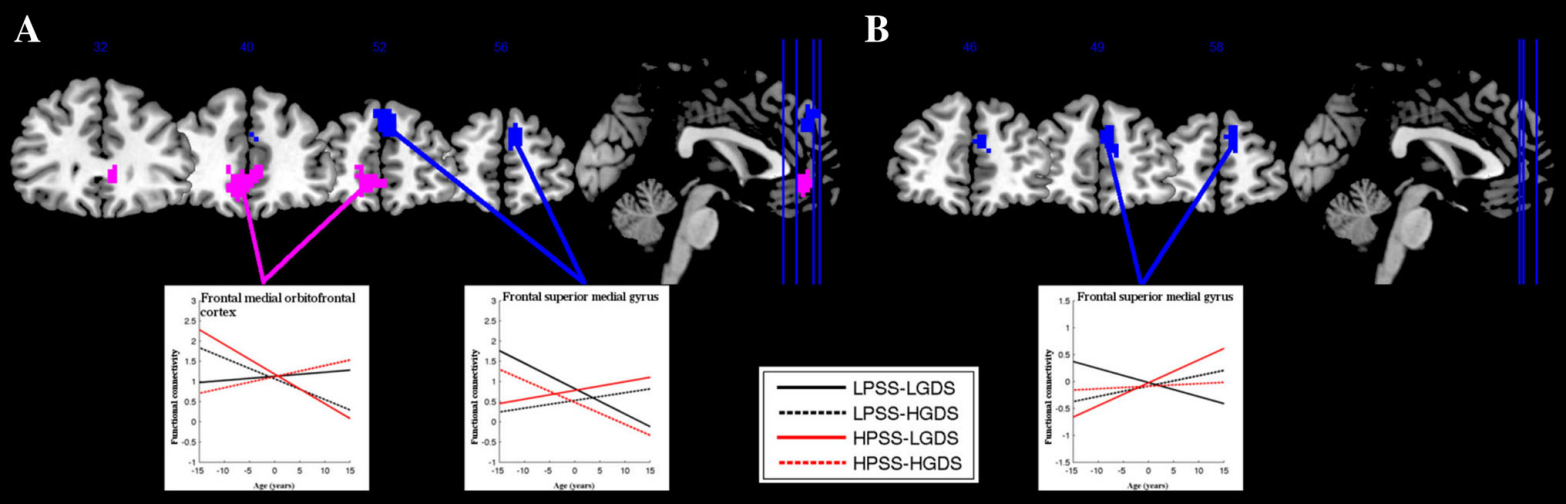
Significant interactions of age × PSS × GDS on the DMN, during resting state (**a**) and task-induced deactivations (**b**). Each graphic line is the regression line between age and functional measures for: low PSS values (LPSS, *black lines*), combined with low GDS (LPSS-LGDS, *solid line*) or with high GDS (LPSS-HGDS, *dotted line*); and high PSS value (HPSS, *red lines*), combined with low GDS (HPSS-LGDS, *solid line*) or high GDS (HPSS-HGDS, *dotted line*)

Finally, we assessed the combined interactions of the three factors: age, stress and mood. During the resting state, the FC of the DMN (in the left frontal medial orbitofrontal cortex and in the right anterior cingulate cortex) maintained the decrease only for the combination low PSS and high GDS scores or with high PSS and low GDS; in fact, in subjects with both low and high PSS and GDS scores, the FC of the DMN increased with age. On the contrary, the FC in the right frontal superior medial gyrus decreased for both low and high PSS and GDS scores, increasing for combined low PSS and high GDS scores or with high PSS and low GDS (Table 4; Fig. 4a). There was no correlation observed between the three factors and the DMN volume (*T* = −0.8164, *p* = 0.42).

During task performance, in the right frontal superior medial gyrus of the DMN, the deactivation decreased with aging for low PSS and GDS scores, increasing for combined low PSS and high GDS scores, high PSS and low GDS, and for combined high scores (Table 5; Fig. 4b).

## Discussion

Recent studies have consistently described the association of the aging process, stress and mood with the brain RSNs, with particular incidence in the DMN (Ferreira and Busatto 2013; Soares et al. 2013a; Kerestes et al. 2013). However, most of the neuroimaging studies focused on the effect of individual factors, precluding the assessment of the complex interaction among various concurrent processes across the lifespan, such as perceived stress and/or variations in depressive mood. Herein, we dissect the association of these life events and how they interact throughout adult life. The present data: (1) replicates the most consistent findings in the literature regarding the association of the adult aging course, stress and major depression with DMN, both during rest and task-induced deactivation; (2) reveals that different stress and mood levels across aging have different correlations with the DMN; and (3) indicates that the interaction between stress and mood states has significant associations with the DMN during the adult lifespan.

In line with previous findings (Damoiseaux et al. 2008; Ferreira and Busatto 2013), an FC decrease in the DMN with increasing age was found, paralleled with a decreased deactivation and a tendency for volumetric reduction (Fjell et al. 2014; Wang et al. 2014). This illustrates the reported difficulty in adaptive switching from a “default mode” to task performance mode in aged individuals and a deficit in cognitive control associated with advancing age (Grady et al. 2010; Sambataro et al. 2010; Anticevic et al. 2012).

The few previous studies investigating the effect of stress on DMN point to a generalized increase of FC under prolonged stress conditions (Soares et al. 2013a, b; Vaisvaser et al. 2013). In this study, and in line with our previous findings in young participants, an increased FC in the more stressed participants (that is, higher PSS scores) was found in the DMN, suggesting augmented self-reflective thoughts and emotional processing (Soares et al. 2013a). In terms of volumetric analysis, a tendency for global DMN atrophy for higher PSS scores was noted, as seen before in younger participants (Soares et al. 2013a), suggesting the existence of both structural and functional reorganizations under stress periods along the lifespan.

A decreased FC in the DMN was also observed in participants with higher GDS scores (that is, more “depressive” mood), along with an increased difficulty to deactivate during cognitive task performance. The results support other findings pointing to a decrease in the connectivity in elderly depressed subjects (Wu et al. 2011; Veer et al. 2010; Andreescu et al. 2011), accompanied by a failure in deactivation during cognitive performance (Sheline et al. 2009), underlying the difficulty in switching between rest and cognitive demanding periods and in connecting internal and external attention in monitoring the external context (Greicius et al. 2003b).

However, the originality of the present work resides on the combined analyses of these factors in the DMN connectivity patterns. In fact, these are likely to be critical for the explanation of the high heterogeneity found in the connectivity of the brain networks, namely the DMN. In fact, while we observed that in the right frontal middle gyrus of the DMN, the FC for low and medium stress levels decreased with age (in line with the typical aging findings; Damoiseaux et al. 2008; Hafkemeijer et al. 2012), we also found that for higher stress levels it increased with aging. These findings show that, as in young stressed subjects (Soares et al. 2013a; Vaisvaser et al. 2013), stress exposure has a similar effect on the DMN across the adult lifespan. The same pattern was observed in the right temporal middle gyrus relative to the mood × age interaction, as the subjects with higher GDS scores also displayed an increased FC in the posterior regions of DMN, which fits with previous observations (Andreescu et al. 2011). Both results point to a more pronounced stress and depressive mood effect (at high levels) over the normal aging pattern on DMN connectivity decline. These apparently contrasting effects observed in the DMN across aging that result from the superimposition of concomitant factors are also observed in deactivation patterns of the DMN, namely in the bilateral frontal superior gyrus, where the deactivation decreases for low stress levels but increases for medium and high levels with age. In fact, for subjects with low stress levels, the typical decline in DMN during aging prevails, whereas for medium and high stress levels there is an increase in the deactivation, suggesting a stress-predominant effect. In the left anterior cingulate cortex, the deactivation increases with age for better mood levels, but decreases for higher depressive levels, confirming the increased difficulty of older and depressed participants to deactivate the DMN (Sheline et al. 2009; Damoiseaux et al. 2008).

The subsequent analysis in this study considered a more complex interaction, as stress and mood are intrinsically connected states that interact over the lifespan (Calabrese et al. 2009). Therefore, we tested for a multifactorial combination of all these factors. Data showed that in the left frontal medial orbitofrontal and right anterior cingulate cortices, the normal aging pattern of FC decrease was only observed for a mixed combination of high and low stress and mood levels. In fact, in these regions of the DMN, the high stress levels lead to an increase in the FC, contrary to the decreases associated with the natural aging process and depression state (Damoiseaux et al. 2008; Andreescu et al. 2011; Wu et al. 2011).

During task performance, specifically in the right frontal superior medial gyrus, there was a typical decrease in deactivation with age, but only in subjects presenting both low stress and depressive mood levels. In fact, we found an increased deactivation with age in subjects presenting with high stress, independently of their mood levels, illustrating again the relevance of the stress effect in deactivation in this region of the DMN.

This study presents limitations that should be addressed. The stress and mood evaluation was based only on neuropsychological scales, without complementary assessment of biological markers. In addition, the study has a cross-sectional design on a sample that includes only older adults which precludes a complete lifespan assessment and, more importantly, a longitudinal characterization.

Nonetheless, to the best of our knowledge, there are no prior reports that evaluate the interaction between stress and mood with the DMN during adult aging. Here, we have shown the critical interaction of stress and mood with the DMN. This multimodal approach may contribute to clarify some literature inconsistencies regarding alterations in FC and deactivation patterns in aging and depression studies (Ferreira and Busatto 2013; Allen et al. 2011; Mowinckel et al. 2012; Kerestes et al. 2013; Wang et al. 2012; Chou et al. 2013). Moreover, we also expect to enlighten the association between DMN activation/deactivation abnormalities and several states that may lead to neuropsychiatric disorders (Meda et al. 2012; Liao et al. 2010; Whitfield-Gabrieli and Ford 2012) that prevail in older individuals.

## References

[CR1] Aiken LS, West SG (1991). Multiple regression: testing and interpreting interactions.

[CR2] Allen EA, Erhardt EB, Damaraju E, Gruner W, Segall JM, Silva RF, Havlicek M, Rachakonda S, Fries J, Kalyanam R, Michael AM, Caprihan A, Turner JA, Eichele T, Adelsheim S, Bryan AD, Bustillo J, Clark VP, Feldstein Ewing SW, Filbey F, Ford CC, Hutchison K, Jung RE, Kiehl KA, Kodituwakku P, Komesu YM, Mayer AR, Pearlson GD, Phillips JP, Sadek JR, Stevens M, Teuscher U, Thoma RJ, Calhoun VD (2011). A baseline for the multivariate comparison of resting-state networks. Front Syst Neurosci.

[CR3] Andreescu C, Wu M, Butters MA, Figurski J, Reynolds CF, Aizenstein HJ (2011). The default mode network in late-life anxious depression. Am J Geriatr Psychiatry.

[CR4] Andrews-Hanna JR (2011). The brain’s default network and its adaptive role in internal mentation. Neuroscientist.

[CR5] Andrews-Hanna JR, Reidler JS, Sepulcre J, Poulin R, Buckner RL (2010). Functional-anatomic fractionation of the brain’s default network. Neuron.

[CR6] Anticevic A, Cole MW, Murray JD, Corlett PR, Wang XJ, Krystal JH (2012). The role of default network deactivation in cognition and disease. Trends Cognit Sci.

[CR7] Arbabshirani MR, Havlicek M, Kiehl KA, Pearlson GD, Calhoun VD (2012). Functional network connectivity during rest and task conditions: a comparative study. Hum Brain Mapp.

[CR8] Bartels A, Zeki S (2005). Brain dynamics during natural viewing conditions—a new guide for mapping connectivity in vivo. Neuroimage.

[CR9] Beckmann CF, DeLuca M, Devlin JT, Smith SM (2005). Investigations into resting-state connectivity using independent component analysis. Philos Trans R Soc Lond B Biol Sci.

[CR10] Bian Y, Pan Z, Hou Z, Huang C, Li W, Zhao B (2012). Learning, memory, and glial cell changes following recovery from chronic unpredictable stress. Brain Res Bull.

[CR11] Buckner RL, Andrews-Hanna JR, Schacter DL (2008). The brain’s default network: anatomy, function, and relevance to disease. Ann N Y Acad Sci.

[CR12] Calabrese F, Molteni R, Racagni G, Riva MA (2009). Neuronal plasticity: a link between stress and mood disorders. Psychoneuroendocrinology.

[CR13] Calhoun VD, Adali T, Pearlson GD, Pekar JJ (2001). A method for making group inferences from functional MRI data using independent component analysis. Hum Brain Mapp.

[CR14] Chou YH, Chen NK, Madden DJ (2013). Functional brain connectivity and cognition: effects of adult age and task demands. Neurobiol Aging.

[CR15] Cohen S, Kamarck T, Mermelstein R (1983). A global measure of perceived stress. J Health Soc Behav.

[CR16] Correa N, Adali T, Li Y, Calhoun V (2005). Comparison of blind source separation algorithms for FMRI using a new Matlab toolbox: GIFT. Proc IEEE Int Conf Acoust Speech Signal Process.

[CR17] Damoiseaux JS, Beckmann CF, Arigita EJ, Barkhof F, Scheltens P, Stam CJ, Smith SM, Rombouts SA (2008). Reduced resting-state brain activity in the “default network” in normal aging. Cereb Cortex.

[CR18] De Vogelaere F, Santens P, Achten E, Boon P, Vingerhoets G (2012). Altered default-mode network activation in mild cognitive impairment compared with healthy aging. Neuroradiology.

[CR19] Dennis AN, Cabeza R, Salthouse FIMCTA (2008). Neuroimaging of healthy cognitive aging. Handbook of aging and cognition.

[CR20] Dias-Ferreira E, Sousa JC, Melo I, Morgado P, Mesquita AR, Cerqueira JJ, Costa RM, Sousa N (2009). Chronic stress causes frontostriatal reorganization and affects decision-making. Science (New York, NY).

[CR21] Ferreira LK, Busatto GF (2013). Resting-state functional connectivity in normal brain aging. Neurosci Biobehav Rev.

[CR22] Fjell AM, McEvoy L, Holland D, Dale AM, Walhovd KB, Alzheimer’s Disease Neuroimaging I (2014). What is normal in normal aging? Effects of aging, amyloid and Alzheimer’s disease on the cerebral cortex and the hippocampus. Prog Neurobiol.

[CR23] Folstein MF, Folstein SE, McHugh PR (1975). “Mini-mental state”. A practical method for grading the cognitive state of patients for the clinician. J Psychiatr Res.

[CR24] Fox MD, Snyder AZ, Vincent JL, Corbetta M, Van Essen DC, Raichle ME (2005). The human brain is intrinsically organized into dynamic, anticorrelated functional networks. Proc Natl Acad Sci USA.

[CR25] Frazier PA, Tix AP, Barron KE (2004). Testing moderator and mediator effects in counseling psychology research. J Couns Psychol.

[CR26] Giugni E, Vadala R, De Vincentiis C, Colica C, Bastianello S (2010). The brain’s default mode network: a mind “sentinel” role?. Funct Neurol.

[CR27] Grady CL, Springer MV, Hongwanishkul D, McIntosh AR, Winocur G (2006). Age-related changes in brain activity across the adult lifespan. J Cogn Neurosci.

[CR28] Grady CL, Protzner AB, Kovacevic N, Strother SC, Afshin-Pour B, Wojtowicz M, Anderson JA, Churchill N, McIntosh AR (2010). A multivariate analysis of age-related differences in default mode and task-positive networks across multiple cognitive domains. Cereb Cortex.

[CR29] Greicius MD, Krasnow B, Reiss AL, Menon V (2003). Functional connectivity in the resting brain: a network analysis of the default mode hypothesis. Proc Natl Acad Sci USA.

[CR30] Greicius MD, Krasnow B, Reiss AL, Menon V (2003). Functional connectivity in the resting brain: a network analysis of the default mode hypothesis. Proc Natl Acad Sci USA.

[CR31] Hafkemeijer A, van der Grond J, Rombouts SA (2012). Imaging the default mode network in aging and dementia. Biochim Biophys Acta.

[CR32] Hansen NL, Lauritzen M, Mortensen EL, Osler M, Avlund K, Fagerlund B, Rostrup E (2014). Subclinical cognitive decline in middle-age is associated with reduced task-induced deactivation of the brain’s default mode network. Hum Brain Mapp.

[CR33] Harrison BJ, Pujol J, Contreras-Rodriguez O, Soriano-Mas C, Lopez-Sola M, Deus J, Ortiz H, Blanco-Hinojo L, Alonso P, Hernandez-Ribas R, Cardoner N, Menchon JM (2011). Task-induced deactivation from rest extends beyond the default mode brain network. PLoS One.

[CR34] Himberg J, Hyvarinen A, Esposito F (2004). Validating the independent components of neuroimaging time series via clustering and visualization. Neuroimage.

[CR35] Huang CC, Hsieh WJ, Lee PL, Peng LN, Liu LK, Lee WJ, Huang JK, Chen LK, Lin CP (2015). Age-related changes in resting-state networks of a large sample size of healthy elderly. CNS Neurosci Ther.

[CR36] Kerestes R, Davey CG, Stephanou K, Whittle S, Harrison BJ (2013). Functional brain imaging studies of youth depression: a systematic review. Neuroimage Clin.

[CR37] Landin-Romero R, McKenna PJ, Salgado-Pineda P, Sarro S, Aguirre C, Sarri C, Compte A, Bosque C, Blanch J, Salvador R, Pomarol-Clotet E (2015). Failure of deactivation in the default mode network: a trait marker for schizophrenia?. Psychol Med.

[CR38] Liao W, Chen H, Feng Y, Mantini D, Gentili C, Pan Z, Ding J, Duan X, Qiu C, Lui S, Gong Q, Zhang W (2010). Selective aberrant functional connectivity of resting state networks in social anxiety disorder. Neuroimage.

[CR39] Marstaller L, Williams M, Rich A, Savage G, Burianova H (2015). Aging and large-scale functional networks: white matter integrity, gray matter volume, and functional connectivity in the resting state. Neuroscience.

[CR40] Mason MF, Norton MI, Van Horn JD, Wegner DM, Grafton ST, Macrae CN (2007). Wandering minds: the default network and stimulus independent thought. Science (New York, NY).

[CR41] Meda SA, Gill A, Stevens MC, Lorenzoni RP, Glahn DC, Calhoun VD, Sweeney JA, Tamminga CA, Keshavan MS, Thaker G, Pearlson GD (2012). Differences in resting-state functional magnetic resonance imaging functional network connectivity between schizophrenia and psychotic bipolar probands and their unaffected first-degree relatives. Biol Psychiatry.

[CR42] Meunier D, Stamatakis EA, Tyler LK (2014). Age-related functional reorganization, structural changes, and preserved cognition. Neurobiol Aging.

[CR43] Mowinckel AM, Espeseth T, Westlye LT (2012). Network-specific effects of age and in-scanner subject motion: a resting-state fMRI study of 238 healthy adults. Neuroimage.

[CR44] Owen AM, McMillan KM, Laird AR, Bullmore E (2005). N-back working memory paradigm: a meta-analysis of normative functional neuroimaging studies. Hum Brain Mapp.

[CR45] Raichle ME, MacLeod AM, Snyder AZ, Powers WJ, Gusnard DA, Shulman GL (2001). A default mode of brain function. Proc Natl Acad Sci.

[CR46] Sambataro F, Murty VP, Callicott JH, Tan HY, Das S, Weinberger DR, Mattay VS (2010). Age-related alterations in default mode network: impact on working memory performance. Neurobiol Aging.

[CR47] Sampaio A, Soares JM, Coutinho J, Sousa N, Goncalves OF (2013). The big five default brain: functional evidence. Brain Struct Funct.

[CR48] Santos NC, Costa PS, Cunha P, Cotter J, Sampaio A, Zihl J, Almeida OF, Cerqueira JJ, Palha JA, Sousa N (2013). Mood is a key determinant of cognitive performance in community-dwelling older adults: a cross-sectional analysis. Age.

[CR49] Sexton CE, Allan CL, Le Masurier M, McDermott LM, Kalu UG, Herrmann LL, Maurer M, Bradley KM, Mackay CE, Ebmeier KP (2012). Magnetic resonance imaging in late-life depression: multimodal examination of network disruption. Arch Gen Psychiatry.

[CR50] Sheline YI, Barch DM, Price JL, Rundle MM, Vaishnavi SN, Snyder AZ, Mintun MA, Wang S, Coalson RS, Raichle ME (2009). The default mode network and self-referential processes in depression. Proc Natl Acad Sci USA.

[CR51] Shirer WR, Ryali S, Rykhlevskaia E, Menon V, Greicius MD (2012). Decoding subject-driven cognitive states with whole-brain connectivity patterns. Cereb Cortex.

[CR52] Soares JM, Sampaio A, Ferreira LM, Santos NC, Marques F, Palha JA, Cerqueira JJ, Sousa N (2012). Stress-induced changes in human decision-making are reversible. Transl Psychiatry.

[CR53] Soares JM, Sampaio A, Ferreira LM, Santos NC, Marques P, Marques F, Palha JA, Cerqueira JJ, Sousa N (2013). Stress impact on resting state brain networks. PLoS One.

[CR54] Soares JM, Sampaio A, Marques P, Ferreira LM, Santos NC, Marques F, Palha JA, Cerqueira JJ, Sousa N (2013). Plasticity of resting state brain networks in recovery from stress. Front Hum Neurosci.

[CR55] Soares JM, Marques P, Magalhaes R, Santos NC, Sousa N (2014). Brain structure across the lifespan: the influence of stress and mood. Front Aging Neurosci.

[CR56] Sousa N, Lukoyanov NV, Madeira MD, Almeida OF, Paula-Barbosa MM (2000). Reorganization of the morphology of hippocampal neurites and synapses after stress-induced damage correlates with behavioral improvement. Neuroscience.

[CR57] Tzourio-Mazoyer N, Landeau B, Papathanassiou D, Crivello F, Etard O, Delcroix N, Mazoyer B, Joliot M (2002). Automated anatomical labeling of activations in SPM using a macroscopic anatomical parcellation of the MNI MRI single-subject brain. Neuroimage.

[CR58] Vaisvaser S, Lin T, Admon R, Podlipsky I, Greenman Y, Stern N, Fruchter E, Wald I, Pine DS, Tarrasch R, Bar-Haim Y, Hendler T (2013). Neural traces of stress: cortisol related sustained enhancement of amygdala-hippocampal functional connectivity. Front Hum Neurosci.

[CR59] Veer IM, Beckmann CF, van Tol MJ, Ferrarini L, Milles J, Veltman DJ, Aleman A, van Buchem MA, van der Wee NJ, Rombouts SA (2010). Whole brain resting-state analysis reveals decreased functional connectivity in major depression. Front Syst Neurosci.

[CR60] Wang L, Hermens DF, Hickie IB, Lagopoulos J (2012). A systematic review of resting-state functional-MRI studies in major depression. J Affect Disord.

[CR61] Wang Y, Chen K, Zhang J, Yao L, Li K, Jin Z, Ye Q, Guo X (2014). Aging influence on gray matter structural associations within the default mode network utilizing bayesian network modeling. Front Aging Neurosci.

[CR62] Whitfield-Gabrieli S, Ford JM (2012). Default mode network activity and connectivity in psychopathology. Annu Rev Clin Psychol.

[CR63] Wu M, Andreescu C, Butters MA, Tamburo R, Reynolds CF, Aizenstein H (2011). Default-mode network connectivity and white matter burden in late-life depression. Psychiatry Res.

[CR64] Yesavage JA, Brink TL, Rose TL, Lum O, Huang V, Adey M, Leirer VO (1982). Development and validation of a geriatric depression screening scale: a preliminary report. J Psychiatr Res.

[CR65] Yuen EY, Wei J, Liu W, Zhong P, Li X, Yan Z (2012). Repeated stress causes cognitive impairment by suppressing glutamate receptor expression and function in prefrontal cortex. Neuron.

[CR66] Zhu X, Wang X, Xiao J, Liao J, Zhong M, Wang W, Yao S (2012). Evidence of a dissociation pattern in resting-state default mode network connectivity in first-episode, treatment-naive major depression patients. Biol Psychiatry.

[CR67] Zuo XN, Kelly C, Adelstein JS, Klein DF, Castellanos FX, Milham MP (2010). Reliable intrinsic connectivity networks: test–retest evaluation using ICA and dual regression approach. Neuroimage.

